# Sex differences in perceived risk and testing experience of HIV in an urban fishing setting in Ghana

**DOI:** 10.1186/s12939-014-0109-z

**Published:** 2014-11-15

**Authors:** Alfred E Yawson, Labi K Appiah, Anita O Yawson, George Bonsu, Simon Aluze-Ele, Nana AK Owusu Amanhyia, Margaret Lartey, Andrew A Adjei, Aaron L Lawson, Curt Beckwith, Awewura Kwara, Timothy Flanigan

**Affiliations:** Department of Community Health, University of Ghana Medical School, College of Health Sciences, P. O. Box 4236, Korle-Bu, Accra, Ghana; Department of Microbiology, Korle-Bu Teaching Hospital, Korle-Bu, Accra, Ghana; Department of Anaesthesia Korle-Bu Teaching Hospital, Korle-Bu, Accra, Ghana; Expanded Programme on Immunization, Public Health Division, Ghana Health Service, Accra, Ghana; Department of Medicine and Therapeutics, University of Ghana Medical School, College of Health Sciences, Korle-Bu, Accra, Ghana; Office of Director of Research, University of Ghana, Legon, Accra, Ghana; Department of Anatomy, University of Ghana Medical School, College of Health Sciences, Korle-Bu, Accra, Ghana; Alpert Medical School of Brown University, The Miriam Hospital, Providence, RI USA

**Keywords:** HIV testing, Sex differences, Perceived risks, Urban community, Ghana

## Abstract

**Introduction:**

Understanding sex differences in willingness to test and testing experience could aid the design of focus interventions to enhance uptake and engagement with care, treatment and support services. This study determined differences in perceived risk of acquiring HIV, willingness to test and HIV testing experience in an urban fishing community.

**Methods:**

A cross-sectional community survey was conducted in 2013 among men and women in two fishing communities (Chorkor and James Town) in Accra. In all, 554 subjects (≥18 years) were involved, 264 in Chorkor and 290 in James Town. Data on *demographic characteristics*, *perceived risk for HIV and willingness to test for HIV* and *testing experience* were collected with a structured questionnaire. Descriptive statistics and Chi square test were used for the analysis at 95% significant level, using SPSS version 21.

**Results:**

Of 554 subjects, 329 (59.4%) were females, and median age was 32 years. Overall, only 91(40.4%) men and 118(35.9%) women perceived themselves to be at risk of acquiring HIV. A significant proportion of women were willing to test for HIV compared to men (86.3% vs. 80.0%, *P* = 0.048). Women were more likely to have ever tested for HIV compared to men (42.2% vs. 28.6%, *P* = 0.001) and more women had tested within 12 months prior to survey than men (49.6% vs. 40.6%, *P* = 0.230). Of the number who had tested for HIV infection, a higher proportion of men tested voluntarily 42(65.6%), while a higher proportion of women tested as part of healthcare service received 96(69.1%); (*P* = 0.001; indicating women vs. men).

**Conclusion:**

Sex differences in risk perception and willingness to test need more focused public education and behaviour change communication strategies to achieve high coverage. Community-based strategies could improve HIV testing among men whilst more access to testing in health settings should be available to women in these communities.

## Introduction

Human immunodeficiency virus (HIV) testing is the gateway into treatment for HIV-infected persons, profoundly reducing AIDS-related morbidity and mortality, as well as new infections [1-3]. In Ghana, HIV prevalence was 1.3% in the adult population in 2013, and 1.9% among pregnant women aged 15–49 years [4]. Although the national prevalence is relatively low compared to other African countries, there are pockets of high prevalence in select geographic areas (e.g. mining and fishing communities) and among select risk groups (commercial sex workers and clients with sexually transmitted infections) [5].

A review of National AIDS Control Programme (NACP) data over the period 2007 to 2010 from all testing avenues in the country shows that the total number of tests conducted represents 16% of the 2010 Ghanaian population [6,7]. Also, the 2008 Ghana Demographic and Health Survey (GDHS) found that 21% of females and 14% of males aged 15–49 years report having ever been tested for HIV [8].

Several studies from Africa have suggested differential use of HIV testing and Counselling (HTC) by sex, with several studies highlighting greater use by females in Southern African countries [9-11] but higher use by males in Ethiopa, Nigeria, Tanzania, and Zambia [12,13].

This high female testing is consistent with previous findings in Ghana, which indicates high willingness for HIV testing among pregnant women in Ghana [14,15]. The limited HIV testing among men is not a new phenomenon in Ghana. Over a decade ago, a study on HIV patients in Ghana found more females were getting tested in hospitals than males [6,16]. Data of the NACP from 2007–2010, shows females comprised 58.2% of those tested for HIV in health facilities across the country, giving a female to male ratio (F: M ratio) of 1.4 [6]. In 2010, a similar F: M ratio of 1.4 for HIV testing was noticed from the community-based HIV testing campaigns. If PMTCT data is included in all HIV tests conducted in 2010, the overall F: M ratio in Ghana increased to 2.8 [6].

HIV testing and counselling (HTC) in Ghana is through three main avenues, the Diagnostic Centres (DCs) which are health facility based locations for checking HIV sero-status. Majority of clients are referred by clinicians for diagnostic testing, this includes persons whom clinical providers are concerned may have HIV, therefore persons at high risk and/or displaying symptoms of infection [5,6]. The second avenue is through Know Your Status Campaigns (KYS) which are HTC activities organized within communities for adults 15 years and above. KYS promote HTC in the general population, irrespective of risk. The third avenue is through Prevention of Mother-to-Child Transmission (PMTCT) services offered as part of reproductive health services for all pregnant women (in the reproductive age group 15–49 years) to know their HIV sero-status [5,6]. HIV testing in Ghana is free, no fees are charged at all [5,6].

Men’s utilization of HTC is important because in many societies men are the heads of households and control decisions and resources that are essential for HIV prevention and care [2,6,8]. To help combat the HIV epidemic in Ghana, increased involvement of men in HIV testing programmes and prevention is critically important.

One critical means of curbing new infections and AIDS-related deaths is by encouraging HIV testing among Ghanaians and linking them to treatment programmes. Significant investments have been made in Ghana to increase access for HIV testing [5,17]. However, the relative lower proportion of men involved in HIV counselling and testing pose a challenge even for the government of Ghana to shoulder the burden surrounding the intervention and treatment of HIV and AIDS related diseases [18]. Men are seen in many societies to be strong and unwilling to display any weakness by seeking healthcare, especially regarding preventive healthcare when they may not be sick at time of seeking care. Women on the other hand are known to engage in far more health promoting behaviours [8,19].

The goal of this study was to determine sex differences in the perceived risk of acquiring HIV, willingness to test and HIV testing experience in an urban fishing setting in Accra, Ghana.

## Methods

### Survey design

A cross-sectional community survey was conducted over a four-week period in September-October 2013, to determine sex differences in perceived risk and testing experience of HIV in an urban fishing setting in Accra, Ghana.

### Survey site

The survey was conducted in (Chorkor in the Ablekuma South Sub-metropolitan area and James Town in the Ashiedu Keteke Sub-metropolitan area) of Accra, Ghana. These urban fishing settings are in the southern part of Accra, (the capital city) with high population densities, large transitory population groups and large youthful populations [20].

### Survey Population and sample size

The survey population were males and females 18 years and above in the communities. Subjects were selected from households within the communities who consented to be part of the survey. The appropriate sample size for the survey was determined by the estimated proportion of community members ever tested for HIV (level of willingness to test), desired level of confidence of 95% and acceptable margin of error of 5%. The Ghana Demographic and Health Survey of 2008, found that 21% of females and 14% of males (aged 15–49 years) have ever been tested for HIV [8]. A 20% willingness to test for HIV among community members was used to estimate the minimum sample size. Accounting for contingencies - non-response, refusals and incomplete information, the minimum sample size was 520 (260 in each community). In all, 554 subjects were involved, 264 in Chorkor and 290 in James Town.

### Sampling methods

The survey employed the modified World Health Organization (WHO) cluster sampling method to select eligible subjects. Each neighbourhood was segmented into four clusters by natural/geographical boundaries. A cluster was chosen by a simple random sampling technique and all eligible subjects within households who consented were included. Houses in the two communities have standard house numbering systems, and households within a house were numbered serially. If more than one household existed within a house, then the first household interviewed was designated as (house number/001)), the second household interviewed was designated as (house number/002), and serially if there were more eligible households within the same house. All households within a selected cluster were eligible for inclusion. One adult member of each household within a house was interviewed, and this continued until the sample size was obtained. The field workers were educated thoroughly on how to assign the codes to households in the two communities.

### Data collection instruments

Data collection tool for the study was a structured questionnaire. Data collected included; *demographic characteristics* (age and sex), *perceived risk for HIV and willingness to test for HIV* (attitudes to HIV testing) and *testing experience* (ever tested or have tested within last 12 months for HIV and reasons for not testing).

The questionnaire was pre-tested at Bukom, a neighbourhood in the Asiedu-Keteke Sub-metropolitan area of Accra with similar characteristics as the two communities for the survey.

### Data handling

All data were treated with a high level of confidentiality. Unique identifiers and codes were employed to de-personify the participants and were used for computer-based data entry. In all cases, study forms, completed questionnaire and other survey documentations were kept securely locked. Computerized records of the survey were kept in locked files. These documents were accessible to the principal investigator only.

### Data analysis

Outcome measures of interest among the demographic and socioeconomic factors (age, sex and location), perceived risk for HIV, willingness to test for HIV and HIV testing experience were analyzed using descriptive statistics (e.g. proportions, frequencies, ratios). Significant differences in categorical outcome measures were analyzed using Chi square test, at the 95% significant level and p-value < 0.05. SPSS version 21 was used for the analysis.

### Ethical issues

Ethical clearance was obtained from the University of Ghana Medical School Ethical and Protocol Review Committee (MS-Et/M.11-P.5.8/2011-2012) and Brown University Institutional Review Board (#1301000744). Trained interviewers administered the questionnaire in the local language of members of the community (i.e. Ga Adangme dialect of the urban fishing community in Accra). Participants in the survey were made to sign a written informed consent form and a waiver of written consent for those participants who were illiterate was obtained. Data was treated with high level of confidentiality and unique identifiers and codes were employed to de-identify the participants and were used for computer-based data entry.

## Results

Overall, 554 subjects were involved in the survey, 225 males and 329 females (as shown in Table 1), given a male to female ratio of 1:1.5. The age of subjects ranged from 18–75 years, with majority of subjects in the age range 21–50 years. The overall mean age ± standard deviation of subjects was 35.2 ± 12.7 years, and was 36.6 ± 9.4 years among the men and 34.4 ± 14.0 years among the women.

**Table 1:** **Age and sex characteristics of subjects in the urban fishing setting, Accra, Ghana**

**Age groups (years)**	**Sex**		**Total**
	**Male**	**Female**	
<20	43 (19.1)	56 (17)	99 (17.9)
21-30	72 (32)	134 (40.7)	206 (37.2)
31-40	56 (24.9)	63 (19.1)	119 (21.5)
41-50	30 (13.3)	39 (11.9)	69 (12.5)
51-60	15 (6.7)	24 (7.3)	39 (7)
> 60	9 (4)	13 (4)	22 (4)
Total	225 (100)	329 (100)	554 (100)

In all, as demonstrated in Table 2, 91 (40.4%) of the men and 118 (35.9%) of women perceived themselves as being at risk of acquiring HIV; relatively higher proportion of men than women perceived themselves as being at risk of acquiring HIV. This difference in risk perception among men and women was however, not statistically significant (p-value = 0.275). Among 134 men in the community who did not perceive themselves to be at risk of HIV, the major reasons given were, they do not engage in casual sexual intercourse, 38 (28.4%); use condoms during sex all the time, 36 (26.9%); and have one faithful partner, 26 (19.4%). Similarly, among 212 women, a relative higher proportion 91 (42.9%) perceived themselves not to be at risk because they have one faithful partner. Other main reasons were that they do not engage in casual sexual intercourse 47 (22.2%) and that they use condoms during sex 42 (19.8%).

**Table 2:** **Perceived risk and willingness to test for HIV among men and women in the urban fishing setting, Accra, Ghana**

**Characteristic**	**Male (%)**	**Female (%)**	**Total (%)**	**P-value** ^*^
**Do you think you may be at risk of acquiring HIV?**				
Yes	91 (40.4)	118 (35.9)	209 (37.7)	
No	134 (59.6)	211 (64.1)	345 (62.3)	0.275
**Total**	**225 (100)**	**329 (100)**	**554 (100)**	
**Reason for not being at risk**				
Have one faithful partner	26 (19.4)	91 (42.9)	117 (33.8)	
Don't engage in sexual intercourse	38 (28.4)	47 (22.2)	85 (24.6)	
Use condoms all the time	36 (26.9)	42 (19.8)	78 (22.5)	**0.001**
I pray for spiritual protection	11 (8.2)	11 (5.2)	22 (6.4)	
I am not sick	14 (10.4)	8 (3.8)	22 (6.4)	
I am a man and strong	0	5 (2.4)	5 (1.4)	
Women are at higher risk	6 (4.5)	3 (1.4)	9 (2.6)	
I decide who to have sex with	3 (2.2)	3 (1.4)	6 (1.7)	
Others	0	2 (0.9)	2 (0.6)	
**Total**	**134 (100)**	**212 (100)**	**346 (100)**	
**Know where to have HIV test done in community**				
Yes	84 (37.3)	109 (33.1)	193 (34.8)	0.275
No	141 (62.7)	22 (66.9)	361 (65.2)	
**Total**	**225 (100)**	**329 (100)**	**554 (100)**	
**An HIV testing site readily accessible**				
Yes	123 (54.7)	195 (59.5)	318 (57.5)	0.264
No	102 (45.3)	134 (40.5)	236 (42.5)	
**Total**	**225 (100)**	**329 (100)**	**554 (100)**	
**Willingness to have HIV test done**				
Yes	180 (80)	284 (86.3)	464 (83.8)	**0.048**
No	45 (20)	45 (13.7)	90 (16.2)	
**Total**	**225 (100)**	**329 (100)**	**554 (100)**	
**Reason for unwillingness to test**				
Don't think am at risk	19 (42.2)	18 (40)	37 (41.1)	
Don't think it is necessary	5 (11.)	8 (17.8)	13 (14.4)	0.962
I am afraid of the outcome	19 (42.2)	16 (35.6)	35 (38.9)	
Others	2 (4.4)	3 (6.7)	5 (5.6)	
Total	**45(100)**	**45 (100)**	**90 (100)**	

*P value for comparing men versus women. Boldface texts under the last column indicate significant P value.

A higher proportion of men than women (37.3% vs. 33.1%; p-value = 0.275) knew where to have HIV test done in the community whereas more women believed an HIV testing site was readily accessible to them (59.5% vs. 54.7%; p-value = 0.264); these differences were not statistically significant though.

A significant higher proportion of women in the two communities were willing to test for HIV compared to men, (86.3% vs. 80%; p-value = 0.048). As illustrated in Table 2, the two main reasons for unwillingness to test among both sexes were that, they did not perceive themselves to be at risk of HIV and being afraid of the outcome of the test.

As indicated in Table 3, a higher proportion of women than men (59% vs. 54.4%; p-value = 0.294) believed it was possible to have a confidential HIV test done in the community. Regarding the actual testing experience of members of the two communities, a statistically significant higher proportion of women 139 (42.2%) had ever tested for HIV compared to men, 64 (28.6%) (p-value = 0.001). Hence close to half of the women had tested while less than a third of the men have. Also, HIV testing within the 12 months prior to the survey indicated a higher proportion of testing among women compared to men (49.6% vs. 40.6%; p-value = 0.230); not statistically significant.

**Table 3:** **HIV testing experience among men and women in the urban fishing setting, Accra, Ghana**

**Characteristic**	**Male (%)**	**Female (%)**	**Total (%)**	**Chi square (p-value)** ^*^
**Confidential HIV testing in community possible**				
Yes	122 (54.4)	194 (59)	316 (57.1)	1.10 (0.294)
No	103 (45.6)	135 (41)	238 (42.9)	
**Total**	**225 (100)**	**329 (100)**	**554 (100)**	
**Ever tested for HIV**				
Yes	64 (28.6)	139 (42.2)	203 (36.7)	**10.73 (0.001)**
No	161 (71.4)	190 (57.8)	351 (63.3)	
**Total**	**225 (100)**	**329 (100)**	**554 (100)**	
**Ever tested for HIV in last 12 months**				
Yes	26 (40.6)	69 (49.6)	95 (46.8)	1.44 (0.230)
No	38 (59.4)	70 (50.4)	108 (53.2)	
**Total**	**64 (100)**	**139 (100)**	**203 (100)**	
**Know results of HIV test**				
Yes	56 (87.5)	115 (66.9)	171 (84.2)	2.32 (0.128)
No	8 (12.5)	24 (16.3)	32 (15.8)	
**Total**	**64 (100)**	**139 (100)**	**203 (100)**	
**Condition for HIV testing**				
Voluntary	42 (65.6)	43 (30.9)	85 (41.9)	**23.71 (0.001)**
Part of health care service	22 (34.4)	96 (69.1)	118(58.1)	
**Total**	**64 (100)**	**139(100)**	**203 (100)**	
**Reason for not testing for HIV**				
Don't think am at risk	53 (32.8)	70 (36.8)	123 (35)	
Don't think it is necessary	10 (6)	18 (9.5)	28 (8)	
I am afraid of the outcome	20 (12.7)	52 (27.4)	72 (20.5)	
I don't have the time	36 (22.4)	29 (15.3)	65 (18.5)	
Never had the opportunity	25 (15.7)	15 (7.9)	40 (11.4)	
I am not sick	7 (4.5)	6 (3.2)	13 (3.7)	
The test is the women	5 (2.2)	0	5 (1.7)	
Will be seen as weak / afraid of the illness	2 (1.5)	0	2 (0.6)	
Other	3(2.2)	0	3 (0.9)	
**Total**	**161 (100)**	**190 (100)**	**351 (100)**	
**HIV testing is useful**				
Yes	193 (85.8)	304 (92.4)	497 (89.7)	**6.35 (0.012)**
No	32 (14.2)	25 (7.6)	57 (10.3)	
**Total**	225 (100)	329 (100)	554 (100)	

*P value for comparing men versus women. Boldface texts under the last column indicate significant P value.

However among those who have ever tested, a higher proportion of men knew their results compared to women (87.5% vs 66.9%). Interestingly, among persons who had ever tested, conditions for testing varied among the sexes. A higher proportion of men did it voluntarily (65.6% vs. 30.9%) whereas most women tested as part of health care received (69.1% vs. 34.4%). This male -female difference in the condition for testing was statistically significant (p-value = 0.001).

Among the 161 men who had never tested for HIV the main reasons were, they did not perceive themselves to be at risk 53 (32.8%); did not have the time 36 (22.4%) and that they have never had the opportunity to do so 25 (15.7%). Similarly, among 172 women, not perceiving themselves to be at risk was the most common reason, 70 (36.6%). Other reasons were fear of outcome of results 52 (27.4%) and not having the time to test 29 (15.3%). It is important to note that only 15 (7.9%) of the women reported never had opportunity to test as reason for not testing.

Although majority of men and women in the two communities agreed that HIV testing was useful, a significant higher proportion of women 304 (92.4%) reported HIV test as useful compared to men 193 (85.8%); (p-value = 0.001).

## Discussion

This community survey of a young population in the age groups at risk for HIV infection found that a majority of both men (60%) and women (64%) perceived themselves as not at risk of acquiring HIV infection. However, women were more willing than men to be tested for HIV infection. Acquisition of HIV has been shown to be more in the young and active segments of the population and HIV testing and counselling (HTC) is the gateway into treatment for HIV-infected persons. Timely initiation of effective HIV treatment can profoundly reduce AIDS-related morbidity and mortality, as well as new infections [1-3,21]. The perception of one being at risk of acquiring HIV should be a critical factor in the decision to seek testing and counselling. Thus, education of these populations is needed to change their perception to enhance voluntary testing or implementation of routine opt out testing in these communities which may be needed to improve testing rates as well as linkage of infected persons to care.

The unwillingness of members of the fishing communities to test for HIV were premised on two main reasons among both sexes , they did not perceive themselves to be at risk of HIV and many were afraid of the outcome of the test. These men felt they were less at risk because they did not engage in casual sexual intercourse, used condoms during sex and had one faithful partner, while the women perceived themselves not to be at risk because they had one faithful partner. Community engagement and increased health and behaviour change communication by the Ghana Health Service through the NACP and other agencies involved in HIV and AIDS activities in Ghana, is imperative. The Ghana Demographic and Health Survey, 2008 indicated high awareness (over 98% among both sexes) but behaviour change and high risk sexual behaviours are still prevalent among sections of the population [5,8]. Sexual networking and the generalized nature of the HIV epidemic in Ghana put all segments of the population at risk irrespective of perceived individual risk [4].

Although nearly 40% of men and 36% of women perceived themselves as being at risk for HIV, the perception of risk of HIV among both groups did not translate into getting to know their HIV status, especially for men. However, a significantly higher proportion of women in the two communities were willing to test for HIV compared to men, (86% vs. 80%) and actual testing experience also indicated a significant higher proportion of women had ever tested for HIV compared to men, (42% vs. 29%). The higher testing among women reflects the national testing patterns from previous published data [6,14,15] as well as testing patterns from the 2008 Demographic and Health Survey in Ghana which indicated a female: male testing ratio of 1.4 [8]. Several studies from other African countries have suggested differential use of HTC by sex, with several studies highlighting greater use by females in southern African countries [9-11]. Thus, interventions to increase testing among men is needed in Ghana and sub-Saharan Africa region as a whole.

Findings from the community assessment is in line with other research which shows women engage in far more health promoting behaviours than men [8,19]. Women in these two communities got tested as part of health care services received compared to men, who had to test voluntarily. Whilst prevention of mother-to-child transmission of HIV (PMTCT) service is able to capture the females, there is no corresponding structure available for capturing the male partners [22]. Unlike their female counterparts, who have PMTCT as an added option, males have limited avenues for HIV testing. The limited HTC use among men in Ghana reinforces the widely reported observation that men are not fully involved in HIV prevention programmes [2,22]. Men’s utilization of HTC is important because in many societies men are the heads of households and control decisions and resources that are essential for HIV prevention and care [2,6].

To increase utility and maximize benefits from investments in HTC in Ghana, the Ministry of Health/Ghana Health Service through the National AIDS/STI Control Programme (NACP) should structure and situate male-focused HTC service programmes in male-dominated occupations, uniformed services, sporting clubs, prisons, male associations in churches/mosques etc. These programmes should also consider and address the mobile nature of male-dominated occupations [6]. In addition, developing routine HIV testing in the casualty and emergency units, inpatient wards and general outpatient departments of health facilities may improve the capture of men.

Improving access to confidential HTC facilities in such urban communities is paramount to the goal of universal coverage for HIV testing. Close to half of men and women (45% and 41% respectively) interviewed in the two communities did not know where to have a confidential HIV test. Most community members believed HIV testing was useful but limited access to a testing site was inhibitory. Evidence from a review of national programme data shows *Know your status (KYS)* organized as community outreach service, outside the health facilities, by the Ghana Health Service/NACP has been shown to attract more people than facility- based testing centres [22]. In addition, KYS seems to be more attractive for men, its disassociation from the health facility makes it easier for them to access services without being seen as weak or sick and is consistent with traditional masculinity ideology that usually cast men as being invulnerable to disease and not needing health care [6,19]. KYS may also be attractive to males because its itinerant nature addresses frequent male mobility [23]. It is important that policy attention is not diverted from this community-based testing programme and resources are needed to target most at risk groups and members of itinerant communities.

### Limitation

Assessment of willingness to test and testing experience were based on subjective recall and responses from the subjects. The survey had no objective way of validating the veracity of information provided by respondents. The pattern of testing among the sexes however, conformed to previous surveys and national level data sources in Ghana.

The study did not provide evidence on linkage to care among those who have tested positive for HIV and how many stayed in care. This was beyond the scope of the survey, however the authors believe emphasis on the care cascade among individuals in the fishing community may be particularly important to evaluate.

## Conclusion

In the urban fishing communities in Accra, a relatively higher proportion of men perceived themselves as being at higher risk of acquiring HIV, however women compared to men, were more willing to test for HIV, had ever tested for HIV and had also tested for HIV within 12 months prior to the survey.

HTC should continue to receive more policy attention and possibly be targeted to more at-risk population groups through population- based programmes such as the Know your status campaigns. Sex differences in risk perception and willingness to test demand more focused public education and behaviour change communication strategies, in addition to structuring male-focused HTC service programmes to improve testing among men.
